# Bioactivity of NANOZR Induced by Alkali Treatment

**DOI:** 10.3390/ijms18040780

**Published:** 2017-04-06

**Authors:** Mariko Nishizaki, Satoshi Komasa, Yoichiro Taguchi, Hiroshi Nishizaki, Joji Okazaki

**Affiliations:** 1 Department of Removable Prosthodontics and Occlusion, Osaka Dental University, 8-1 Kuzuhahanazonocho, Hirakata, Osaka 573-1121, Japan; komasa-s@cc.osaka-dent.ac.jp (S.K.); nisizaki@cc.osaka-dent.ac.jp (H.N.); joji@cc.osaka-dent.ac.jp (J.O.); 2 Department of Periodotology, Osaka Dental University, 8-1 Kuzuhahanazonocho, Hirakata, Osaka 573-1121, Japan; taguchi@cc.osaka-dent.ac.jp

**Keywords:** zirconia/alumina nano-composite, bioactivity, alkali treatment

## Abstract

In recent years, zirconia has been a recognized implant material in clinical dentistry. In the present study, we investigated the performance of an alkali-modified ceria-stabilized tetragonal ZrO_2_ polycrystalline ceramic-based nanostructured zirconia/alumina composite (NANOZR) implant by assessing surface morphology and composition, wettability, bovine serum albumin adsorption rate, rat bone marrow (RBM) cell attachment, and capacity for inducing bone differentiation. NANOZR surfaces without and with alkali treatment served as the control and test groups, respectively. RBM cells were seeded in a microplate with the implant and cultured in osteogenic differentiation medium, and their differentiation was evaluated by measuring alkaline phosphatase (ALP) activity, osteocalcin (OCN) production, calcium deposition, and osteogenic gene expression. The alkali-treated NANOZR surface increased ALP activity, OCN production, calcium deposition, and osteogenesis-related gene expression in attached RBM cells. These data suggest that alkali treatment enhances the osteogenesis-inducing capacity of NANOZR implants and may therefore improve their biointegration into alveolar bone.

## 1. Introduction

Various materials and surface modification methods have been developed to improve implant osseointegration and reduce healing time in clinical dentistry [1,2,3,4]. Oral implants made of titanium and its alloys have been shown to function well for many years [5]. Surface characteristics of the implant material affect the rate and extent of osseointegration [6,7]; there is increasing evidence that surface-modified materials are highly effective for promoting cell adhesion, growth, and osteogenic differentiation [8].

In recent years, zirconia has been a recognized implant material for various reasons [9,10,11]. Firstly, zirconia is more compatible with esthetic requirements since the dark color of titanium can show through the pinkish hue of the cervical gingiva, especially in patients with a thin gingival biotype [12]. In addition, high titanium concentrations in tissue have been reported in the vicinity of titanium oral implants and in regional lymph nodes, suggesting that titanium is a sensitinogen in some individuals [13]. Moreover, products of titanium particle corrosion may provoke host reactions [14] and are thus a potential health hazard. These findings have prompted some patients to request metal-free dental reconstructions. Zirconia ceramics exhibit high strength and fracture toughness [15,16] and are suitable for transgingival implant components, although their behavior over the long term has not been investigated in detail.

Ceria-stabilized tetragonal zirconia polycrystals (Ce-TZP) and alumina polycrystals (Al_2_O_3_) have been combined to generate a ceria-stabilized tetragonal ZrO_2_ polycrystalline ceramic-based nanostructured zirconia/alumina composite (NANOZR), in which Al_2_O_3_ nanoparticles are dispersed among Ce-TZP granules [16,17,18]. This material is highly resistant to low-temperature degradation and is especially useful for load-bearing applications such as dental implants, exhibiting greater flexural strength and fracture toughness and more than twice the cyclic fatigue strength of 3 mol % yttria-stabilized tetragonal zirconia polycrystals (3Y-TZP) [19].

The detailed mechanism of osseointegration is still unclear. However, osseointegration properties of biomaterials can be assessed by examining the behavior of osteoblasts on the implant surface [20,21]. Various studies have compared adhesion and differentiation of rat bone marrow (RBM) cells on titanium and zirconia surfaces [20,21,22]. Each kind of method has its own advantages. In a recent study, Li showed that this layer induces apatite formation for osseointegration by alkali treatment. In some research, acid treatment led to the lamination of the oxide layer of titanium and zirconia surface as well as changing the surface roughness [23]. Furthermore, sand blast changed the surface roughness of materials of titanium and zirconia surface. Our team has a track record of alkali treatment to titanium and titanium alloy surface and we have reported the clinical utility of this treatment in a previous study [24]. In our study, we used the innovative condition to prepare the titanium metal surfaces in 10 M NaOH aqueous solution at 30 °C, which produces a roughened nanoscale surface. Alkali-modified titanium surface was shown to promote osteogenic differentiation in RBM cells [24,25,26]. Material surface properties and structures play important roles in protein adsorption, which can in turn influence cell behavior. We speculated that applying the above treatment to the NANOZR surface could improve the rate of osseointegration.

To test this hypothesis, the present study compared the performance of alkali-modified NANOZR surfaces by evaluating surface morphology and composition, wettability, bovine serum albumin (BSA) adsorption rate, RBM cell attachment, and capacity of inducing bone differentiation. The findings provide a basis for improving the success rate of zirconia and titanium dental implants.

## 2. Results

### 2.1. Sample Preparation

**Figure 1:**
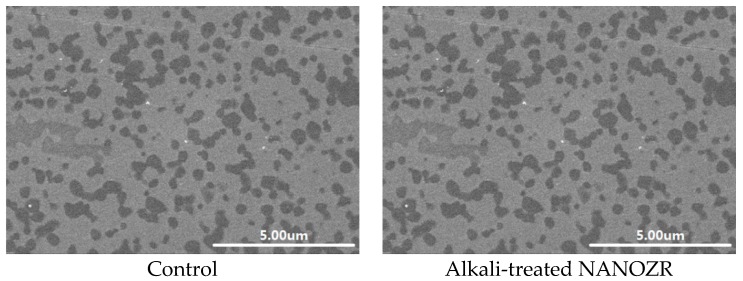
SEM micrographs of control and test groups.

**Figure 2:**
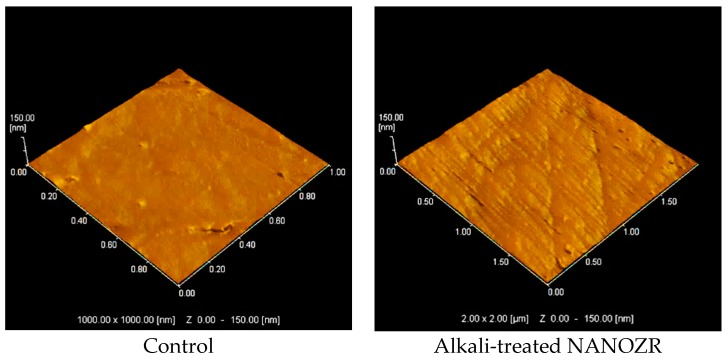
Scanning probe (SP) micrographs of control and test groups.

**Figure 3:**
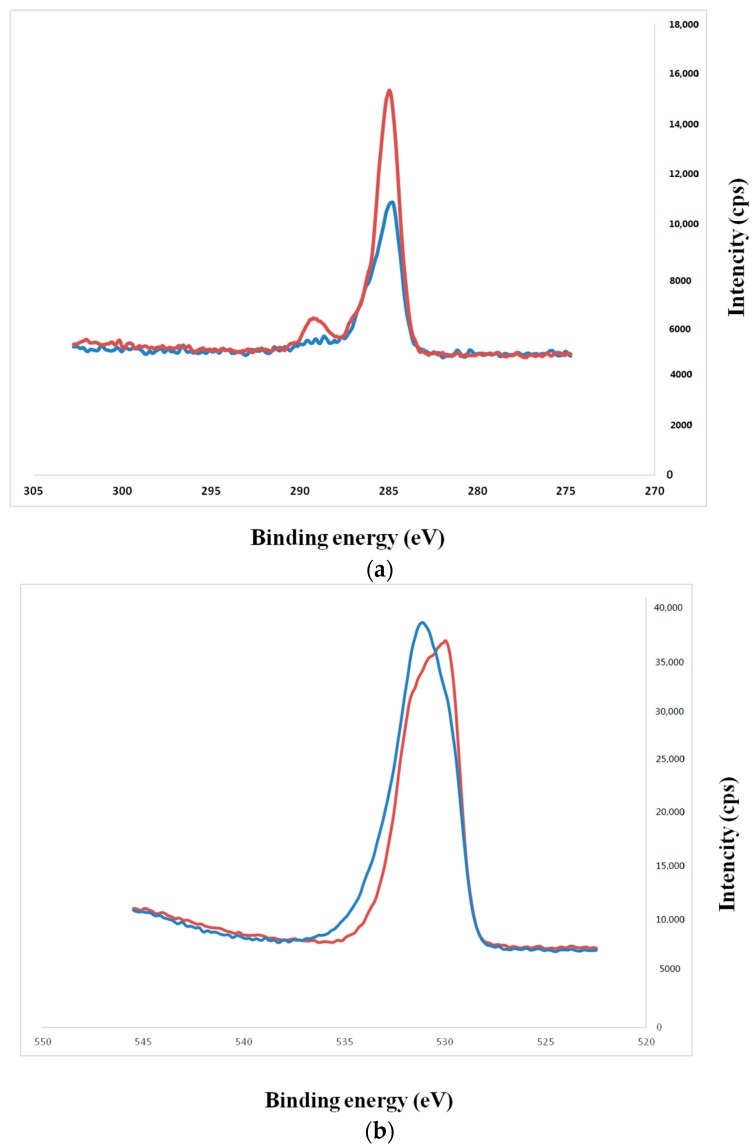

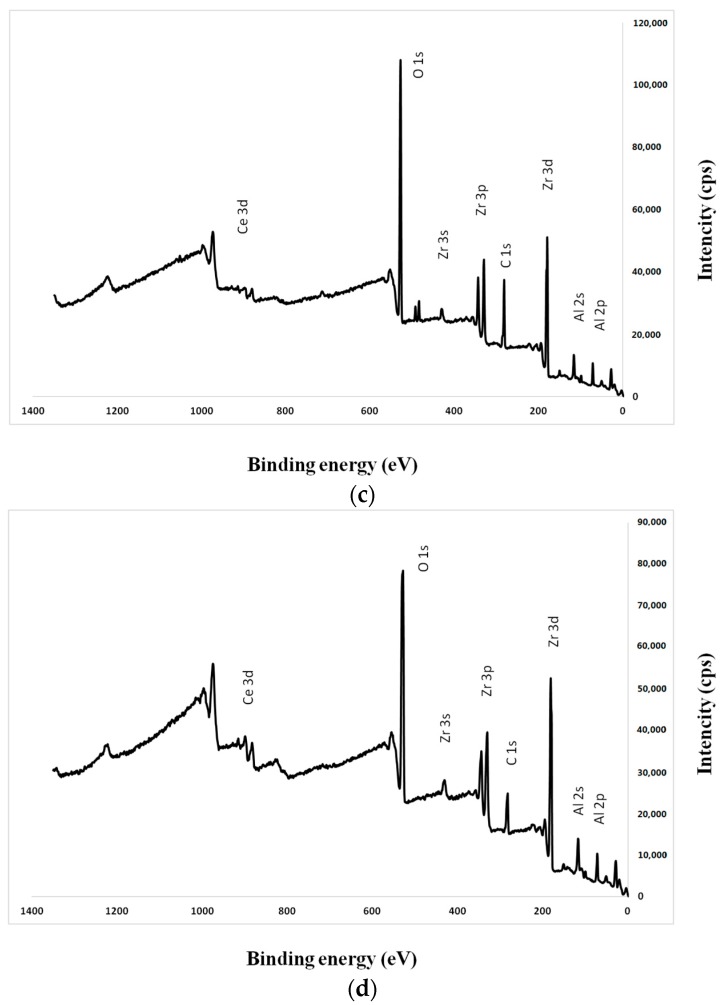
(**a**) C1s XPS spectrum of the NANOZR surface. (Red line: control, blue line: alkali-treated NANOZR); (**b**) C1s XPS spectrum of the NANOZR surface. (red line: control, blue line: alkali-treated NANOZR); (**c**) Wide scan of the XPS spectrum of the alkali**-**treated NANOZR surface; (**d**) Wide scan of the XPS spectrum of the untreated NANOZR surface.

**Figure 4:**
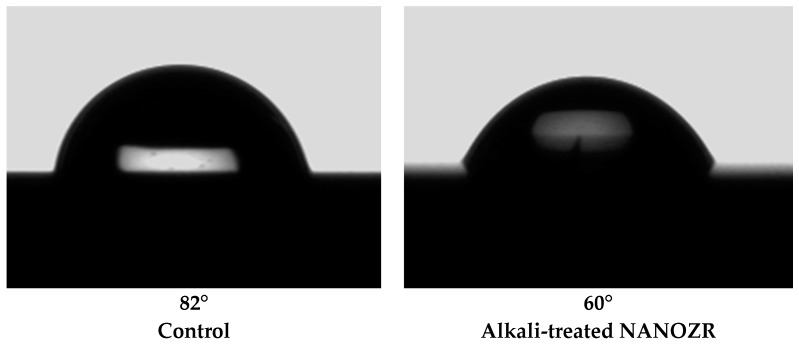
Contact angles in control and test groups.

Scanning electron microscopy (SEM) observations revealed no structural changes on the NANOZR surface after immersion in NaOH solution (Figure 1); however, a roughened surface was observed by scanning probe microscopy (SPM) (Figure 2). The Ra of the alkali-treated NANOZR surface was 1.8 nm, which was higher than that of the untreated surface (Ra = 0.9 nm). In the X-ray photoelectron spectroscopy (XPS) analysis, the intensity of the O1s peaks was increased while that of the C1s peaks were decreased by NaOH treatment. The Na peak was not assigned. At narrow scan of O1s, two types of O atoms were mixed, and peaks of O atoms (OH type) were assigned by NaOH treatment (Figure 3). There was a significant difference between contact angles measured for the alkali-treated NANOZR and untreated NANOZR disks (Figure 4).

### 2.2. Protein Adsorption

**Figure 5:**
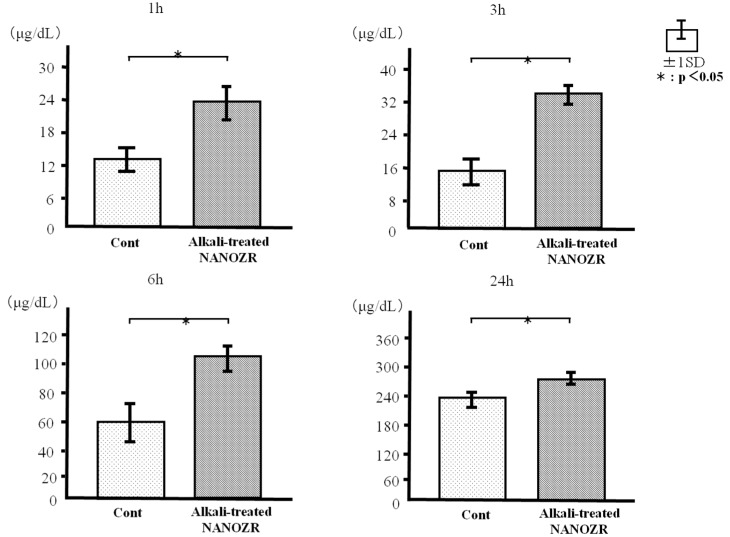
Protein adsorption in control and test groups.

We measured the amount of BSA adsorbed on the alkali-modified and untreated NANOZR surfaces after incubation for 1, 3, 6, and 24 h. The alkali-treated NANOZR surface showed greater BSA adsorption at each time point than the untreated surface (Figure 5).

### 2.3. Cell Adhesion and Morphology

**Figure 6:**
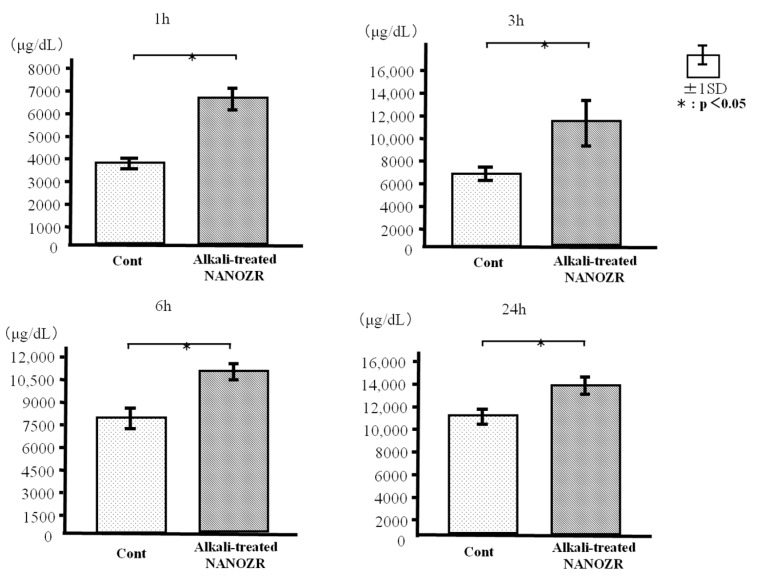
Cell adhesion in control and test groups.

The adhesion of RBM cells on the specimens was evaluated after 1, 3, 6, and 24 h of incubation. Alkali-treated NANOZR showed greater RBM cell adhesion at each time point than the untreated NANOZR surface (Figure 6).

**Figure 7:**
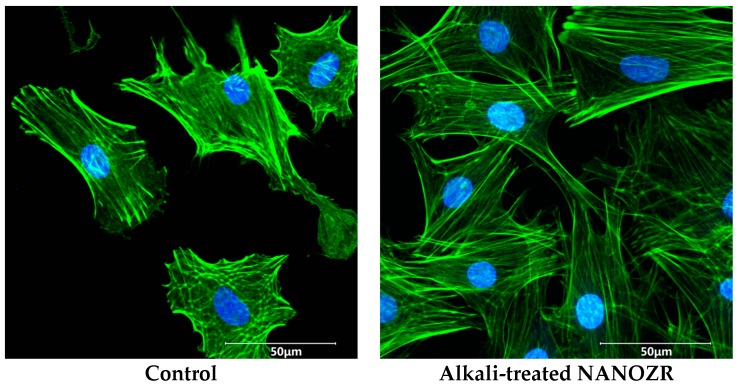
Morphology of rat bone marrow (RBM) cells in the control and test groups after culturing for 24 h. Actin filaments (**green**) were labeled with Alexa Fluor 488-phalloidin and nuclei (**blue**) were stained with 4′,6-diamidino-2-phenylindole (DAPI).

Cell morphology was evaluated by Alexa Fluor 488-phalloidin and 4′,6-diamidino-2-phenylindole (DAPI) staining after 24 h of incubation. Compared to RBM cells attached to the untreated NANOZR surface, those on alkali-modified NANOZR showed greater F-actin expression and more filopodia and lamellipodia (Figure 7).

### 2.4. Quantitative Real-Time (qRT-)PCR Analysis of Osteogenesis-Related Gene Expression

**Figure 8:**
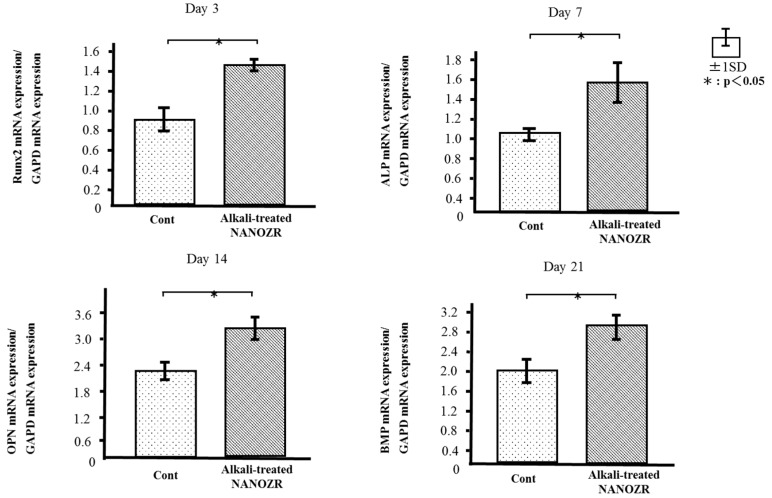
Quantitative real-time (qRT)-PCR analysis of osteogenesis-related gene expression in control and test groups.

The mRNA expression levels of osteogenesis-related genes including *alkaline phosphatase* (*ALP*), *runt-related transcription factor* (*Runx2*), *bone morphogenetic protein* (*BMP*), and *osteopontin* (*OPN*) in RBM cells grown on the different surfaces for 3, 7, 14, and 21 days were assessed by qRT-PCR. The levels of all genes were upregulated in cells grown on alkali-treated NANOZR surfaces as compared to those grown on untreated NANOZR (Figure 8).

### 2.5. ALP Activity

**Figure 9:**
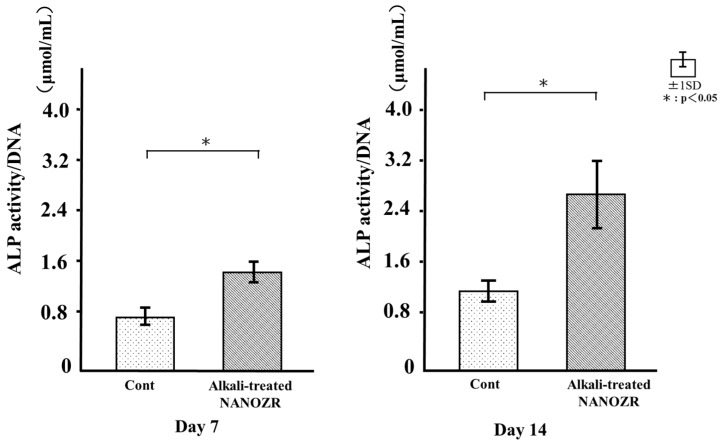
Alkaline phosphatase (ALP) activity in control and test groups.

ALP activity was observed in RBM cells grown on the different substrates for 7 days, and was found to increase with time. There were significant differences in ALP activity between alkali-treated and untreated NANOZR surfaces at 7 and 14 days (Figure 9), with higher levels in the former than in the latter group (Figure 9).

### 2.6. Osteocalcin (OCN) Production

**Figure 10:**
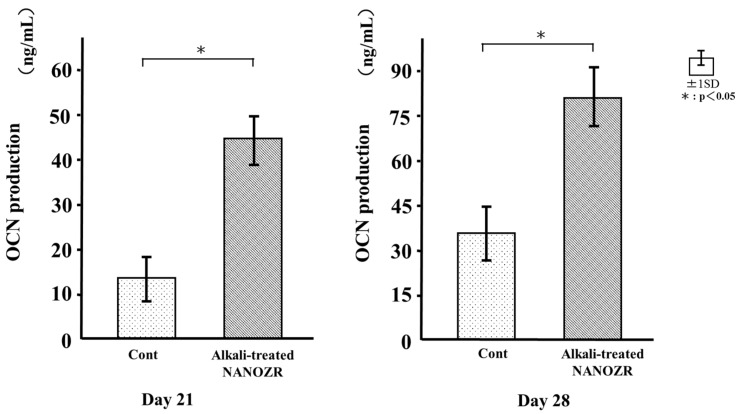
Osteocalcin (OCN) production in control and test groups.

We measured OCN levels in the culture supernatant of RBM cells grown on the different substrates for 21 and 28 days. The supernatant of cells grown on the alkali-treated NANOZR surface showed higher levels of OCN at both time points than that of control cultures (Figure 10).

### 2.7. Mineralization

**Figure 11:**
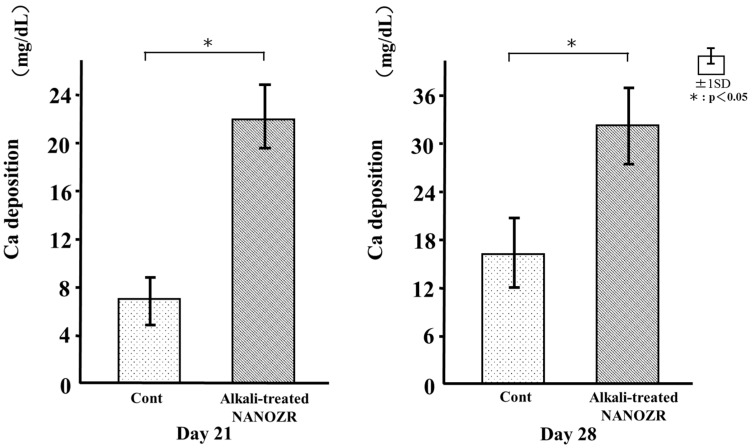
Calcium deposition in control and test groups.

Calcium deposition in cells cultured on the different surfaces for 21 and 28 days was assessed as a measure of osteogenic differentiation. Mineralization was greater in cells grown on the alkali-treated NANOZR surface than in those on the untreated surface (Figure 11).

## 3. Discussion

The present study investigated whether RBM cells respond differently to NANOZR implants that have been subjected to surface modification by alkali treatment. We found that adhesion and osteogenic differentiation—including calcium deposition in the extracellular matrix—were increased in cells grown on the alkali-treated as compared to the untreated, polished NANOZR surface. These results suggest that similar to alkali-modified titanium, alkali treatment of NANOZR promotes RBM cell adhesion and osteogenic differentiation.

The potential for using zirconia as a dental implant material has been previously investigated because of its mechanical properties, chemical stability, and aesthetic advantages [27,28]. Zirconia implants have shown better osseointegration without causing inflammation, demonstrating biocompatibility [29,30,31]. The surface morphology and chemical composition of zirconia is also superior to that of pure titanium. Zirconia-based NANOZR [17,18] exhibits greater flexural strength and fracture toughness than conventional 3Y-TZP, making it a suitable material for use in dental implants. In a recent study, we presented the response of rat bone marrow cells to three groups—alkali treated NANOZR, untreated NANOZR, and alkali treated titanium surface—at the congress of the 30th Japan Association of Oral Rehabilitation in Japan [32]. There are no significant differences in osteogenic activity between alkali treated NANOZR and alkali treated titanium surface. Thus, we evaluated osteogenic activity between alkali treated NANOZR and untreated NANOZR in this study.

Surface properties and composition are major determinants of the cellular response to implants [33]. For instance, a roughened surface has been shown to improve early cell attachment to the implant surface [34]. Accordingly, we found that the Ra of the alkali-treated NANOZR surface was higher than that of the untreated specimen (1.8 vs. 0.9 nm). A study investigating the osteoblastic response to different zirconia surface topographies found that cell adhesion was higher on rough than on smooth surfaces [35,36]. Our results indicate that the surface topography of zirconia is an important factor for improving the initial attachment of osteoblasts. NANOZR has a characteristic topography consisting of several 10–100 nm ZrO_2_ particles trapped within the Al_2_O_3_ grains. Nano-sized grains have been shown to enhance protein adsorption, osteoblast adhesion, and osteogenic differentiation [37]. Nano-scale ZrO_2_ and Al_2_O_3_ grains also affect surface energy and consequently, cellular response. A change of surface wettability corresponds to a low contact angle, which promotes cell adhesion to a surface. Our XPS analysis revealed that the intensity of O1s peaks was increased while those of the Zr3d and C1s peaks were decreased by NaOH treatment, resulting in a smaller contact angle. In the narrow scan of O1s, two types of O atoms were mixed, and peaks of O atoms (OH type) were assigned by NaOH treatment. Li showed that the Zr–OH layer was similar to the Ti-OH layer, and this layer induced apatite formation for osseointegration. [23]. In general, zirconia surfaces—including the NANOZR surface—are covered with a zirconia oxide layer that can react with NaOH solution. We observed that the hydrated zirconium oxide layer formed an amorphous structure whose thickness increased with NaOH treatment time. Titanium gel layers charged with Na+ ion form a titanate hydrogel layer; in contrast, the zirconia in NANOZR forms a Na-free zirconia hydrogel layer. We found that the C1s and corresponding shoulder peak (ascribed to hydrocarbon contamination) were decreased along with the atomic percentage of carbon in alkali-treated NANOZR, indicating that brief exposure to alkaline agents can reduce surface carbon content. C and O derived from organic materials or metal oxides have been detected on the material surface by XPS analysis [38]; in our study, the decrease in C1s and increase in O1s peak intensity of the alkali-treated NANOZR surface was associated with increased RBM cell adhesion and osteogenic differentiation. Uchida et al. prepared zirconium metal at 95 °C for 24 h successfully. The modification method used here is useful and easily accomplished because the incubation required in NaOH is at room temperature, as this study demonstrates that low and high temperature methods increase RBM cell adhesion and osteogenic differentiation.

All implant surfaces are immediately covered with a layer of protein from biological fluids, creating an interface that modulates the cascade of cellular responses [39]. Albumin is the most abundant plasma protein and inhibits the adsorption of proteins that can stimulate inflammation and bacterial colonization. In this study, alkali-treated NANOZR showed higher BSA adsorption than the untreated specimen. The adsorption of proteins or ions may act as a bridge between the nanostructured surface and cells. The formation of actin filaments—which are involved in cell adhesion and bone differentiation—was increased on the alkali-treated as compared to the untreated NANOZR surface, as determined by phalloidin staining, which revealed that RBM cells attached by spreading across the roughened surface via actin-based filopodia and lamellipodia.

The alkali-treated NANOZR surface increased ALP activity, OCN production, calcium deposition, and osteogenesis-related gene expression in RBM cells. ALP activity is a biochemical marker of the osteoblast phenotype at early stages of differentiation as well as of bone formation and osteoblast activity. OCN is a non-collagenous, glutamate-rich, polypeptide bone matrix protein with a molecular weight of about 5800 kDa. Osteoblasts produce and incorporate OCN into the bone matrix; OCN is released into the circulation from the matrix during bone resorption and as such, is considered as a marker of bone turnover rather than of bone formation. We observed differences in the expression levels of osteoblast-specific markers between alkali-treated and untreated implant surfaces. Runx2 and BMP are key transcription factors mediating osteoblast differentiation; the observed upregulation in Runx2 and BMP as well as ALP and OPN levels in RBM cells grown on the alkali-treated surface reflects its greater capacity for inducing osteogenic differentiation.

## 4. Materials and Methods

### 4.1. Sample Preparation

NANOZR disks 15 mm in diameter and 1.5 mm thick (Panasonic Health Care, Tokyo, Japan) were used in this study. The NANOZR disk surface was first polished with a diamond wheel (#400) using a grinding machine (Surface Grinder PSG52DX; Okamoto Machine Tool Works, Annaka, Japan) followed by a diamond particle slurry paste (6–12, 2–6, and 0–1 μm) using a polishing machine (Compact Desktop Lapping System EJ-3801N; Engis Japan, Yokohama, Japan). The disks were immersed in 10 M aqueous NaOH at 30 °C for 24 h. The solution in each flask was replaced with distilled water (200 mL) until the solution reached a conductivity of 5 μS/cm. Samples were then dried at room temperature. One hundred fifty eight disks were divided into NANOZR and alkali-treated NANOZR groups.

### 4.2. Surface Characterization

The surface topography of samples was qualitatively evaluated by SEM (S-400; Shimadzu, Kyoto, Japan) and SPM (SPM-9600; Shimadzu). The composition of the coating was analyzed by XPS (Kratos Analytical Axis Ultra DLD electron spectrometer; Kratos Instruments, Manchester, UK) using a monochromatic Al Kα X-ray source. Argon-ion etching was performed for 2 min (evaporation rate 5 nm/min) on each sample to remove surface contaminants. Contact-angle measurements with ultrapure water were performed at room temperature using a video contact-angle measurement system (VSA 2500 XE; AST Products, Tokyo, Japan).

### 4.3. Protein Adsorption

BSA fraction V (Pierce Biotechnology, Rockford, IL, USA) was used as a model protein. Protein solution (300 μL, 1 mg/mL protein in saline) was pipetted onto each sample. After incubation for 1, 3, 6, or 24 h at 37 °C, non-adherent proteins were removed and mixed with bicinchoninic acid (Pierce Biotechnology) at 37 °C for 1 h. The removed and total amounts of inoculated BSA were quantified using a microplate reader at 562 nm. The rate of albumin adsorption was calculated as the percentage of BSA adsorbed to samples relative to the total amount of BSA in solution.

### 4.4. Cell Culture

Five RBM cells were isolated from the femurs of 8-week-old Sprague-Dawley rats. The study protocol was performed under the Guidelines for Animal Experimentation at Osaka Dental University (approval no. 16-08001). Rats were euthanized with 4% isoflurane, and hind limbs bones were aseptically excised. The proximal end of the femur and distal end of the tibia were clipped. A 21-gauge needle (Terumo, Tokyo, Japan) was inserted into the hole in the knee joint of each bone, and the marrow was flushed from the shaft with Eagle’s minimal essential medium (Wako Pure Chemical Industries, Osaka, Japan) supplemented with 10% fetal bovine serum (Invitrogen/Life Technologies, Carlsbad, CA, USA), penicillin (500 U/mL) (Cambrex Bio Science Walkersville, Walkersville, MD, USA), streptomycin (500 μg/mL) (Cambrex Bio Science Walkersville), and Fungizone (1.25 μg/mL) (Cambrex Bio Science Walkersville). The resultant marrow pellet was dispersed by trituration, and cell suspensions from all bones were combined in a centrifuge tube. RBM cells were cultured in 75-cm^2^ Falcon culture flasks (BD Biosciences, Franklin Lakes, NJ, USA) in culture medium. When cells reached confluence, they were removed from the flasks by trypsinization, washed twice with phosphate-buffered saline (PBS), resuspended in culture medium, and seeded at a density of 4 × 10^4^/cm^2^ in 24-well Falcon tissue culture plates (BD Biosciences) containing test or control NANOZR disks, and cultured at 37 °C in a humidified atmosphere of 5% CO_2_/95% air.

### 4.5. Cell Adhesion

Cell adhesion was evaluated with the CellTiter-Blue Cell Viability Assay kit (Promega, Madison, WI, USA) according to the manufacturer’s protocol. Briefly, RBM cells were seeded on samples at a density of 4 × 10^4^/cm^2^ and allowed to attach for 1, 3, 6, and 24 h. At predetermined time points, non-adherent cells were removed by rinsing with PBS. CellTiter-Blue Reagent (50 μL) and PBS (250 μL) were then added to each well. After incubation at 37 °C for 1 h, the solution was removed from the tissue culture plates and 100 μL was transferred to a new Falcon 96-well tissue culture plate (BD Biosciences). The optical densities at 560 and 590 nm of the remaining solution were measured. The difference between the two optical densities was defined as the proliferation value.

### 4.6. Cell Morphology

Three samples were washed with PBS, fixed by incubation with 4% paraformaldehyde solution for 20 min at room temperature, and permeabilized with 0.2% (*v*/*v*) Triton X-100 for 30 min at room temperature after 24 h. Cells were incubated with Blocking One reagent (Nacalai Tesque, Kyoto, Japan) for 30 min at room temperature and then stained with Alexa Fluor 488-phalloidin (Invitrogen/Life Technologies) and DAPI at 37 °C in the dark for 1 h. F-actin and cell nuclei were visualized by confocal laser scanning microscopy (LSM700; Carl Zeiss, Oberkochen, Germany).

### 4.7. qRT-PCR Analysis

Total RNA was extracted from cells and 1 μg was used to synthesize cDNA using a High-Capacity cDNA Archive kit (Applied Biosystems, Foster City, CA, USA) after 3, 7, 14, and 21 days. *ALP*, *Runx2*, *BMP*, and *OPN* mRNA expression was investigated by qRT-PCR on a StepOne Plus Real-Time RT-PCR system (Applied Biosystems). Taqman Fast Universal PCR Master Mix (10 μL), 1 μl of the primer probe set (20× Taqman Gene Expression Assays), sample cDNA (2 μL), and diethylpyrocarbonate-treated water (Nippongene, Toyama, Japan; 7 μL) were added to each well of a Fast 96-well Reaction Plate (0.1-mL well volume; Applied Biosystems). The plate was subjected to 40 reaction cycles of 95 °C for 1 s and 60 °C for 20 s. Target gene expression level was calculated with the 2^−ΔΔ*C*t^ method relative to the negative control group.

### 4.8. ALP Activity

RBM cells were washed with PBS and lysed with 200 μL of 0.2% Triton X-100 (Sigma-Aldrich, St. Louis, MO, USA). The lysate was transferred to a microcentrifuge tube containing a 5-mm hardened steel ball after 7 and 14 days of culture. Tubes were agitated on a shaker (Mixer Mill Type MM 301; Retsch GmbH, Haan, Germany) at 29 Hz for 20 s to homogenize the sample. ALP activity was measured using the Alkaline Phosphatase Luminometric enzyme-linked immunosorbent assay (ELISA) kit (Sigma-Aldrich) according to the manufacturer’s protocol. The reaction was terminated with 3 N NaOH to a final concentration of 0.5 N NaOH and *p*-nitrophenol production was determined by measuring absorbance at 405 nm on a 96-well microplate reader (SpectraMax M5; Molecular Devices, Sunnyvale, CA, USA). DNA content was measured with the PicoGreen dsDNA Assay kit (Invitrogen/Life Technologies) according to the manufacturer’s protocol. The amount of ALP was normalized to the amount of DNA in the cell lysate.

### 4.9. OCN ELISA Analysis

After 21 and 28 days of culture, sandwich ELISA was used to determine OCN levels directly in the cell culture supernatant with a commercial kit (Rat Osteocalcin ELISA kit; DS Pharma Biomedical Co., Osaka, Japan) according to the manufacturer’s instructions.

### 4.10. Mineralization

Mineralization was assessed using a Calcium E-test kit (Wako Pure Chemical Industries). At each point (21 or 28 days of culture), 1 mL Calcium E-test reagent and 2 mL buffer were added to the 50 μL of the collected medium. It was measured the absorbance of the reaction products at 610 nm by using a 96-well microplate reader (Falcon). The concentration of calcium ions was calculated according to the manufacturer’s instructions.

### 4.11. Statistical Analysis

All samples were prepared in triplicate. Data are presented as the mean ± standard deviation. In all analyses, statistical significance was determined with the paired two-tailed Student’s *t*-test. A *p*-value < 0.05 was considered statistically significant.

## 5. Conclusions

In conclusion, our study revealed that modifying a NANOZR implant surface by alkali treatment promoted RBM cell attachment and osteogenic differentiation. We consider that the change in surface roughness and surface wettability as well as the decrease in C1s and increase in O1s peak intensity of the alkali-treated NANOZR surface was associated with increased RBM cell adhesion and osteogenic differentiation in our study. Although further studies are needed to confirm these effects in vivo, our findings nonetheless indicate that the osseointegration of zirconium-based implants can be improved by surface nanotopographical modification.

## Abbreviations

3Y-TZP	yttria-stabilized tetragonal zirconia polycrystals
Al_2_O_3_	alumina polycrystals
ALP	alkaline phosphatase
BMP	bone morphogenetic protein
BSA	bovine serum albumin
Ce-TZP	ceria-stabilized tetragonal zirconia polycrystals
DAPI	4′,6-diamidino-2-phenylindole
NANOZR	ceria-stabilized tetragonal ZrO_2_ polycrystalline ceramic-based nanostructured zirconia/alumina composite
OCN	osteocalcin
OPN	osteopontin
PBS	phosphate-buffered saline
RBM	rat bone marrow
Runx2	runt-related transcription factor 2
SEM	scanning electron microscopy
SPM	scanning probe microscopy
TiO_2_	titania
TNS	titanium nanosheets
XPS	X-ray photoelectron spectroscopy
