# Correction: Mariko Nishizaki, et al. Bioactivity of NANOZR Induced by Alkali Treatment. *Int. J. Mol. Sci.* 2017, 18, 780

**DOI:** 10.3390/ijms18092009

**Published:** 2017-09-19

**Authors:** Mariko Nishizaki, Satoshi Komasa, Yoichiro Taguchi, Hiroshi Nishizaki, Joji Okazaki

**Affiliations:** 1 Department of Removable Prosthodontics and Occlusion, Osaka Dental University, 8-1 Kuzuhahanazonocho, Hirakata, Osaka 573-1121, Japan; komasa-s@cc.osaka-dent.ac.jp (S.K.); nisizaki@cc.osaka-dent.ac.jp (H.N.); joji@cc.osaka-dent.ac.jp (J.O.); 2 Department of Periodotology, Osaka Dental University, 8-1 Kuzuhahanazonocho, Hirakata, Osaka 573-1121, Japan; taguchi@cc.osaka-dent.ac.jp

We would like to submit the following correction to the published paper [1]. The reason for the correction is that the data provided in Figure 1 were represented by the same picture. When we contributed the data, the pictures were correct. After review, we put a scale bar in the picture of test group, the picture was replaced the picture of control group with by mistake. For such reason, the test group data provided in Figure 1 should be replaced with the correct picture. The SEM data of test group was changed, but for the SEM data of the control and test group, there were no structural changes on the NANOZR surface after immersion in NaOH solution. Figure 1 should be replaced:

In addition, there was a written error: (**b**) C1s XPS spectrum of the NANOZR surface. (red line: control, blue line: alkali-treated NANOZR) of Figure 3 was corrected to (**b**) Zr3d XPS spectrum of the NANOZR surface. (red line: control, blue line: alkali-treated NANOZR).

These changes have no material impact on the conclusions of our paper. The manuscript will be updated and the original will remain online on the article webpage. We apologize for any inconvenience caused to our readers.

## Figures and Tables

**Figure 1:**
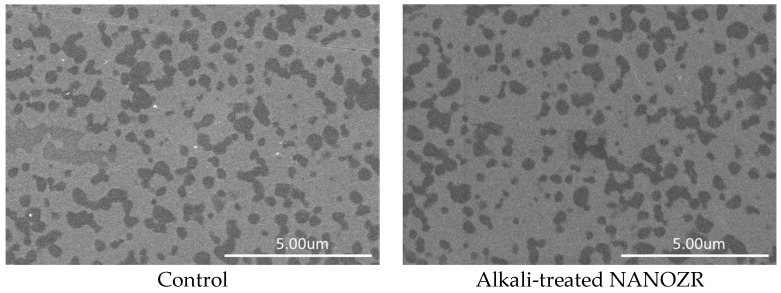
SEM micrographs of control and test groups.

## References

[1] Nishizaki M., Komasa S., Taguchi Y., Nishizaki H., Okazaki J. (2017). Bioactivity of NANOZR Induced by Alkali Treatment. Int. J. Mol. Sci..

